# Differential Risk Factors for Hematoma Expansion in Deep and Lobar Intracerebral Hemorrhage

**DOI:** 10.1007/s12028-025-02218-z

**Published:** 2025-02-04

**Authors:** Kangwei Zhang, Baoqing Yang, Lai Wei, Xiang Zhou, Fushi Han, Jinxi Meng, Xingyu Zhao, Bo Zhang, Daxiao Chen, Peijun Wang

**Affiliations:** 1 Department of Medical Imaging, Tongji Hospital, Tongji University School of Medicine, Shanghai, China; 2 Institute of Medical Imaging Artificial Intelligence, Tongji University School of Medicine, Shanghai, China; 3 Department of Cardiopulmonary Rehabilitation, Shanghai Yangzhi Rehabilitation Hospital (Shanghai Sunshine Rehabilitation Center), Tongji University School of Medicine, Shanghai, China

**Keywords:** Intracerebral hemorrhage, Hematoma extension, Risk factors, Prognosis, Hematoma location

## Abstract

**Background:**

Understanding the risk factors for hematoma expansion (HE) in different regions of intracerebral hemorrhage (ICH) can help in the development of more accurate HE prediction tools and in implementing more effective clinical treatment interventions. This study aims to investigate the risk factors for HE in patients with lobar and deep ICH.

**Methods:**

A retrospective analysis was conducted on 558 cases of primary supratentorial ICH from Tongji Hospital Affiliated to Tongji University. Patients were categorized into lobar ICH and deep ICH groups. Differential analysis of ICH characteristics at different locations was performed, followed by subgroup analysis based on HE occurrence. Binary logistic regression was used to identify independent risk factors for HE in each group.

**Results:**

Among the 404 patients with ICH who underwent follow-up noncontrast computed tomography (NCCT) scans, the proportion with HE was similar in the deep ICH group (23.2%) and the lobar ICH group (22.7%). Binary logistic regression analysis revealed that fluid level (odds ratio [OR] 4.77, 95% confidence interval [CI] 1.74–13.06), admission Glasgow Coma Scale score (OR 0.87, 95% CI 0.80–0.96), and time from onset to NCCT examination (OR 0.84, 95% CI 0.75–0.94) were independently associated with HE in the deep ICH group. In the lobar ICH group, irregular shape (OR 4.96, 95% CI 1.37–18.01) and fibrinogen level (OR 0.42, 95% CI 0.21–0.86) were significant risk factors.

**Conclusions:**

Fluid level, low admission Glasgow Coma Scale score, and shorter time from onset to NCCT are independent predictors of HE in deep ICH, whereas irregular shape and low fibrinogen levels are independent predictors of HE in lobar ICH. These findings are of great significance for elucidating the mechanisms underlying HE in different locations of ICH and for developing precise predictive models of HE.

**Supplementary Information:**

The online version contains supplementary material available at 10.1007/s12028-025-02218-z.

## Introduction

Spontaneous intracerebral hemorrhage (ICH) is characterized by its acute onset and high rates of mortality and disability [1]. Hematoma expansion (HE) is associated with poor prognosis and serves as a significant marker of disease progression and a critical target for clinical treatment in ICH [2].

Previous studies have explored various risk factors for HE, including imaging, clinical history, and laboratory markers [3–8]. However, there has been no comprehensive summary of the risk factors for HE in different regions of ICH. Morotti et al. have found that subarachnoid hemorrhage can be used to predict the occurrence of HE in lobar ICH [9]. Kuohn et al. and Roh et al. have noted differences in clinical characteristics and HE outcomes between deep and lobar ICH [10–12]. However, they did not conduct a subgroup analysis on deep ICH and lobar ICH to further investigate the risk factors for HE occurrence in different brain regions. A deeper understanding of the risk factors for HE in different brain hemorrhage locations can clarify the mechanisms behind HE in these areas. This knowledge is crucial for developing more precise artificial intelligence–based prediction tools for HE and for implementing more effective clinical treatment interventions. Comparing the clinical characteristics and prognostic differences between different regions of ICH can help deepen our understanding of the pathogenesis of ICH.

The primary objective of this study was to investigate the risk factors for HE in deep ICH and lobar ICH. Additionally, we compared the differences in clinical history, imaging markers, and laboratory indices between different regions of ICH.

## Methods

### Study Participants

**Fig. 1:**
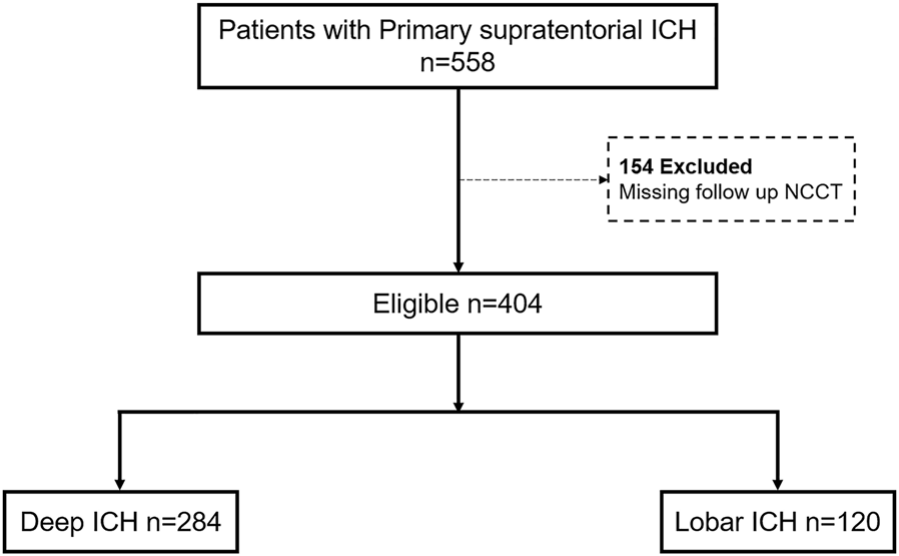
Patient selection flowchart. ICH, intracerebral hemorrhage; NCCT noncontrast computed tomography

A retrospective analysis was conducted on the clinical data of 558 consecutive patients with primary supratentorial ICH from Tongji Hospital Affiliated to Tongji University admitted between November 2018 and March 2024. All patients with ICH meeting the inclusion criteria during this period were included. Each patient underwent a head computed tomography (CT) scan within 24 h of symptom onset and a follow-up scan within 72 h. The selection and grouping process of the patients is illustrated in Fig. 1.

The studies involving human participants were reviewed and approved. Informed consent was not mandatory for participation in this study, in compliance with national legislation and institutional guidelines.

### Inclusion Criteria

Inclusion criteria were as follows: (1) diagnosis of primary supratentorial ICH confirmed by the initial noncontrast CT (NCCT) scan within 24 h of symptom onset and a second NCCT scan within 72 h, (2) access to high-quality baseline and follow-up NCCT images, and (3) age ≥ 18 years.

### Exclusion Criteria

Exclusion criteria were as follows: (1) ICH caused by trauma, vascular malformation, tumor, or other secondary reasons; (2) primary intraventricular hemorrhage; (3) missing imaging data; (4) follow-up NCCT after hematoma evacuation surgery; and (5) hemorrhagic transformation of ischemic stroke.

### Image Acquisition, Processing, and Analysis

NCCT scans were acquired with a thickness of 5 mm for axial reconstruction, following specific NCCT acquisition protocols for each region. Hematoma volume and density were measured using 3D Slicer software (available at https://www.slicer.org/) [13]. NCCT markers and the van Swieten scale were analyzed by two neuroradiologists with more than 5 years of clinical experience who were blinded to clinical outcomes. In case of disagreement, a chief attending physician made the final judgment. HE was the primary outcome, defined as an absolute increase in hematoma volume > 6 mL or a relative increase in volume from baseline to follow-up NCCT > 33%. ICH location was assessed on baseline NCCT and classified as lobar ICH (involving the cortex and cortico-subcortical junction) and deep ICH (involving the thalamus, basal ganglia, internal capsule, and periventricular white matter).

### Clinical Variables

A total of 41 potential risk factors for HE were collected through outpatient follow-up and review of hospital medical records and any available medical records, including clinical history, imaging markers, and laboratory tests. Clinical information included age, sex, smoking, drinking, history of diabetes, coronary atherosclerosis, atrial fibrillation, hypertension, stroke history, time from onset to NCCT, rate of bleeding, admission systolic and diastolic blood pressure, admission Glasgow Coma Scale (GCS) score, anticoagulant therapy, hemostatic therapy, and antiplatelet therapy. Baseline laboratory parameters such as creatine kinase, blood glucose, fibrinogen, C-reactive protein (CRP), D-dimer, activated partial thromboplastin time, prothrombin time/international normalized ratio (PT/INR), leukocyte count, neutrophil count, lymphocyte count, neutrophil-to-lymphocyte ratio (NLR), plasma B-type natriuretic peptide (BNP), and others were recorded. Imaging parameters included initial CT hematoma size, mean density, midline shift, intraventricular hemorrhage, van Swieten scale (the variable was transformed into a dichotomous variable based on whether its value exceeded 1), hypodensities sign, blend sign, island sign, black hole sign, irregular shape, fluid level, and subarachnoid hemorrhage.

### Statistical Analysis

Data analysis was conducted using SPSS 20.0. Continuous variables were expressed as means ± standard deviations (SDs) or medians(interquartile ranges [IQRs]), whereas categorical variables were presented as counts (percentages). Distribution normality was assessed using the Kolmogorov–Smirnov test. For normally distributed variables, means ± SDs were used, and *t*-tests were employed for group comparisons. Comparisons of continuous variables between groups were conducted using the Mann–Whitney *U*-test, and for binary data, the Pearson χ^2^ test was used. Significant variables (*P* < 0.05) were selected for further multivariate analysis. Variables were tested for multicollinearity, and any with multicollinearity were excluded. A binary logistic regression model was constructed, and the correlation between multiple variables and HE was explored. Forward stepwise regression was employed for variable selection. To mitigate the risk of Type I errors, we applied the Bonferroni correction to the statistical significance threshold, setting the critical *P* value at 0.05/*n* (where *n* represents the number of independent variables being evaluated). The model’s fit was assessed using the Hosmer–Lemeshow goodness-of-fit test.

## Results

### Demographic Data of Patients

Among the 404 patients with ICH who met the inclusion criteria, 120 (29.7%) were diagnosed with lobar ICH, and 284 (70.3%) were classified as having deep ICH. Table 1 presents a comparison of differences between the two groups. The proportion of patients with HE in the deep ICH group (66, 23.2%) was slightly higher than that in the lobar ICH group (27, 22.5%), but the difference was not statistically significant (*P* = 0.87). Patients with lobar ICH had a significantly higher age (median 71.50 years; IQR 64.00–84.00 years) compared to patients with deep ICH (median 64.00 years; IQR 53.00–71.00 years). The time from symptom onset to NCCT (h) in patients with deep ICH (median 2.98; IQR 1.57–5.10) was shorter than that in patients with lobar ICH (median 4.36; IQR 2.14–8.98). The admission GCS score was significantly lower in patients with deep ICH (median 12.00; IQR 9.00–13.00) than in patients with lobar ICH (median 14.00; IQR 11.00–15.00). Patients with lobar ICH (50, 41.7%) exhibited more severe leukoaraiosis compared to those with deep ICH (77, 27.1%). Both systolic and diastolic blood pressures were significantly higher in patients with deep ICH than in patients with lobar ICH. The prevalence of coronary atherosclerosis was significantly higher in patients with lobar ICH (82, 68.3%) compared to patients with deep ICH (145, 51.1%). A higher proportion of patients with lobar ICH (23, 19.2%) had been on antiplatelet therapy before the onset of symptoms compared to patients with deep ICH (32, 11.3%). Based on NCCT imaging findings, subarachnoid hemorrhage, fluid level, irregular shape, blend sign, and island sign were significantly more common in the lobar ICH group than in the deep ICH group. Conversely, midline shift and intraventricular hemorrhage were more prevalent in  the deep ICH group than in the lobar ICH group. The hematoma volume (ml) was significantly larger in  the lobar ICH group (median 20.73; IQR 8.46–37.93) compared to the deep ICH group (median 12.84; IQR 6.94–22.46). In terms of laboratory test results, PT/INR, CRP, NLR, and BNP were all significantly higher in patients with lobar ICH than in patients with deep ICH.

**Table 1:** Lobar vs. deep ICH baseline characteristics

Variable	All ICH (*n* = 404)	Lobar ICH (*n* = 120)	Deep ICH (*n* = 284)	*P* value
HE (> 6 mL or 33%), *n* (%)	93 (23.0)	27 (22.5)	66 (23.2)	0.87
Male,* n* (%)	278 (68.8)	77 (64.2)	201 (70.8)	0.19
Age, median (IQR), y	65.00 (55.25–75.00)	71.50 (64.00–84.00)	64.00 (53.00–71.00)	< 0.001
Time from onset to NCCT, median (IQR), h	3.29 (1.69–6.16)	4.36 (2.14–8.98)	2.95 (1.56–5.13)	< 0.001
Admission GCS score, median (IQR)	12.00 (9.00–14.00)	14.00 (11.00–15.00)	12.00 (9.00–13.00)	< 0.001
van Swieten scale, median (IQR)	1.00 (0.00–2.00)	1.00 (0.00–3.00)	1.00 (0.00–2.00)	0.04
van Swieten scale > 1, *n* (%)	127 (31.4)	50 (41.7)	77 (27.1)	0.004
Admission systolic pressure, median (IQR), mmHg	180.00 (160.00–193.00)	170.00 (154.00–190.00)	185.50 (165.00–199.50)	< 0.001
Admission diastolic pressure, median (IQR), mmHg	102.00 (88.00–116.00)	99.00 (82.00–110.50)	104.00 (92.25–120.00)	0.001
Smoking, *n* (%)	102 (25.2)	29 (24.2)	73 (25.7)	0.75
Drinking, *n* (%)	88 (21.8)	19 (15.8)	69 (24.3)	0.06
Diabetes, *n* (%)	72 (17.8)	25 (20.8)	47 (16.5)	0.30
Coronary sclerosis, *n* (%)	227 (56.2)	82 (68.3)	145 (51.1)	0.001
Atrial fibrillation, *n* (%)	26 (6.4)	10 (8.3)	16 (5.6)	0.31
Hypertension, *n* (%)	310 (76.7)	89 (74.2)	221 (77.8)	0.43
Stroke, *n* (%)	73 (18.1)	26 (21.7)	47 (16.5)	0.22
Anticoagulant therapy, *n* (%)	86 (21.3)	23 (19.2)	63 (22.2)	0.50
Hemostatic therapy, *n* (%)	295 (73.0)	90 (75.0)	205 (72.2)	0.56
APT, *n* (%)	55 (13.6)	23 (19.2)	32 (11.3)	0.03
Subarachnoid hemorrhage, *n* (%)	67 (16.6)	51 (42.5)	16 (5.6)	< 0.001
Fluid level, *n* (%)	45 (11.1)	23 (19.2)	22 (7.7)	0.001
Irregular shape, *n* (%)	230 (56.9)	84 (70.0)	146 (51.4)	0.001
Hypodensities, *n* (%)	145 (35.9)	48 (40.0)	97 (34.2)	0.26
Blend sign, *n* (%)	120 (29.7)	54 (45.0)	66 (23.2)	< 0.001
Island sign, *n* (%)	72 (17.8)	34 (28.3)	38 (13.4)	< 0.001
Black hole sign, *n* (%)	56 (13.9)	21 (17.5)	35 (12.3)	0.17
MLS, *n* (%)	145 (35.9)	23 (19.2)	122 (43.0)	< 0.001
IVH, *n* (%)	126 (31.2)	21 (17.5)	105 (37.0)	< 0.001
Volume, median (IQR), ml	13.83 (7.26–26.73)	20.73 (8.46–37.93)	12.84 (6.94–22.46)	< 0.001
Density, mean ± SD, hu	58.83 ± 4.97	58.65 ± 5.46	58.91 ± 4.76	0.10
Rate of bleeding, median (IQR), ml/h	4.24 (1.64–9.62)	4.16 (1.62–10.92)	4.25 (1.67–9.40)	0.86
Creatine kinase, median (IQR), U/L	90.00 (61.50–140.50)	88.00 (55.50–132.20)	91.00 (63.00–143.50)	0.24
Blood glucose, median (IQR), mmol/L	7.26 (6.26–8.79)	7.23 (6.17–9.19)	7.31 (6.30–8.79)	0.75
Fibrinogen, median (IQR), g/L	2.64 (2.27–3.18)	2.62 (2.29–3.32)	2.65 (2.27–3.16)	0.24
D-dimer, median (IQR), mg/L	0.49 (0.25–1.25)	0.55 (0.35–1.15)	0.46 (0.24–1.26)	0.09
APTT, median (IQR), s	27.10 (25.70–29.10)	27.50 (25.80–29.40)	27.05 (25.55–28.80)	0.27
PT-INR, median (IQR)	0.94 (0.90–0.99)	0.95 (0.92–1.02)	0.93 (0.89–0.98)	0.001
CRP, median (IQR), mg/L	2.17 (0.87–5.83)	3.07 (1.07–9.13)	1.85 (0.84–5.01)	0.01
Leukocyte count, median (IQR), 10^9^/L	8.48 (6.71–10.69)	8.73 (7.07–10.41)	8.46 (6.50–11.03)	0.40
Neutrophile granulocyte count, median (IQR), 10^9^/L	6.20 (4.37–8.85)	6.25 (4.85–8.79)	6.15 (4.06–8.89)	0.19
Lymphocyte count, median (IQR), 10^9^/L	1.40 (0.95–1.97)	1.25 (0.89–1.88)	1.46 (1.00–2.01)	0.09
NLR, median (IQR)	4.32 (2.41–8.27)	5.47 (2.96–9.14)	3.93 (2.25–7.92)	0.03
BNP, median (IQR), pg/ml	60.80 (35.30–116.30)	67.10 (44.10–152.00)	55.75 (32.23–106.50)	0.01

APT, antiplatelet therapy; APTT, activated partial thromboplastin time; BNP, B-type natriuretic peptide; CRP, C-reactive protein; GCS, Glasgow coma scale; HE, hematoma expansion; ICH, intracerebral hemorrhage; IQR, interquartile range; IVH, intraventricular hemorrhage; MLS, midline shift; NCCT, noncontrast computed tomography; NLR, neutrophil-to-lymphocyte ratio; PT-INR, prothrombin time/international normalized ratio

Tables 2 and 3 present the results of a comparative analysis between the two subgroups (HE vs. non-HE) in the deep ICH and lobar ICH patient cohorts, respectively. In the deep ICH cohort, the time from symptom onset to the first NCCT examination (h) was significantly shorter in the HE group (median 1.71; IQR 0.93–3.46) compared to the non-HE group (median 3.34; IQR 1.90–5.99), whereas no significant difference was observed between the two groups in the lobar ICH cohort. In the deep ICH cohort, patients in the HE group had a significantly low GCS score at admission (median 10.50; IQR 7.00–13.00) compared to those in the non-HE group (median 12.00; IQR 9.00–14.00), whereas no significant difference was noted in the lobar ICH cohort. The proportion of patients receiving anticoagulant and antiplatelet therapy was significantly higher in the HE group compared to the non-HE group in the deep ICH cohort, whereas no significant difference was observed in the lobar ICH cohort. In the lobar ICH cohort, the proportion with subarachnoid hemorrhage was significantly higher in the HE group (16, 59.3%) compared to the non-HE group (35, 37.6%), whereas this difference was not observed in the deep ICH cohort. Among imaging markers based on NCCT, the proportions of patients showing fluid level, hypodensities, irregular shape, and blend sign were significantly higher in the HE group compared to the non-HE group in the deep ICH cohort, whereas irregular shape and blend sign were significantly higher in the HE group and fluid level and hypodensities sign showed no significant difference between the two groups in the lobar ICH cohort. The initial hematoma volume (ml) was significantly larger in the HE group (median 30.12; IQR 12.70–45.05) compared to the non-HE group (median 19.13; IQR 7.22–34.72) in the lobar ICH cohort, whereas this difference was not significant in the deep ICH cohort. The rate of bleeding (ml/h) was significantly higher in the HE group (median 8.54; IQR 3.67–19.96) compared to the non-HE group (median 3.59; IQR 1.51–7.44) in the deep ICH cohort, whereas no significant difference was observed in the lobar ICH cohort. In terms of laboratory test differences, the deep ICH HE group showed significantly lower D-dimer levels, higher lymphocyte counts, and a higher NLR compared to the non-HE group. In the lobar ICH cohort, the HE group had significantly lower fibrinogen levels, higher neutrophil counts, and a higher NLR compared to the non-HE group. These differences were statistically significant.

**Table 2:** Univariate analysis of clinical baseline characteristics in the two deep ICH cohorts

Variable	Non-HE (*n* = 218)		HE (*n* = 66)	*P* value
Male, *n* (%)	154 (70.6)	47 (71.2)		0.93
Age, mean ± SD, y	62.78 ± 14.03	64.52 ± 13.96		0.38
Time from onset to NCCT, median (IQR), h	3.34 (1.90–5.99)	1.71 (0.93–3.46)		< 0.001
Admission GCS score, median (IQR)	12.00 (9.00–14.00)	10.50 (7.00–13.00)		0.001
van Swieten scale, median (IQR)	1.00 (0.00–2.00)	1.00 (0.00–1.25)		0.62
van Swieten scale > 1, *n* (%)	61 (28.0)	16 (24.2)		0.55
Admission systolic pressure, median (IQR), mmhg	181.00 (162.00–196.00)	190.00 (169.50–200.00)		0.10
Admission diastolic pressure, median (IQR), mmhg	103.00 (92.00–120.00)	109.00 (93.50–120.00)		0.41
Smoking, *n* (%)	53 (24.3)	20 (30.3)		0.33
Drinking, *n* (%)	51 (23.4)	18 (27.3)		0.52
Diabetes, *n* (%)	35 (16.1)	12 (18.2)		0.68
Coronary sclerosis, *n* (%)	109 (50.0)	36 (54.5)		0.52
Atrial fibrillation, *n* (%)	11 (5.0)	5 (7.6)		0.44
Hypertension, *n* (%)	170 (78.0)	51 (77.3)		0.90
Stroke, *n* (%)	34 (15.6)	13 (19.7)		0.43
Anticoagulant therapy, *n* (%)	41 (18.8)	22 (33.3)		0.01
Hemostatic therapy, *n* (%)	157 (72.0)	48 (72.7)		0.91
APT, *n* (%)	18 (8.3)	14 (21.2)		0.004
Subarachnoid hemorrhage, *n* (%)	12 (5.5)	4 (6.1)		0.86
Fluid level, *n* (%)	11 (5.0)	11 (16.7)		0.002
Irregular shape, *n* (%)	105 (48.2)	41 (62.1)		0.047
Hypodensities, *n* (%)	64 (29.4)	33 (50.0)		0.002
Blend sign, *n* (%)	44 (20.2)	22 (33.3)		0.03
Island sign, *n* (%)	27 (12.4)	11 (16.7)		0.37
Black hole sign, *n* (%)	26 (11.9)	9 (13.6)		0.71
MLS, *n* (%)	87 (39.9)	35 (53.0)		0.06
IVH, *n* (%)	78 (35.8)	27 (40.9)		0.45
Volume, median (IQR), ml	12.52 (6.78–21.37)	15.24 (9.01–28.58)		0.07
Density, mean ± SD, hu	58.90 ± 4.54	58.93 ± 5.46		0.97
Rate of bleeding, median (IQR), ml/h	3.59 (1.51–7.44)	8.54 (3.67–19.96)		< 0.001
Creatine kinase, median (IQR), U/L	88.00 (61.00–152.25)	97.00 (69.00–126.00)		0.87
Blood glucose, median (IQR), mmol/L	7.31 (6.26–8.79)	7.29 (6.41–9.10)		0.59
Fibrinogen, median (IQR), g/L	2.66 (2.27–3.17)	2.52 (2.27–3.12)		0.34
D-dimer, median (IQR), mg/L	0.55 (0.24–1.26)	0.30 (0.19–0.95)		0.02
APTT, median (IQR), s	27.00 (25.40–28.63)	27.55 (25.95–29.80)		0.14
PT-INR, median (IQR)	0.93 (0.89–0.97)	0.96 (0.89–1.00)		0.06
CRP, median (IQR), mg/L	1.86 (0.82–4.97)	1.81 (0.86–5.46)		0.78
Leukocyte count, median (IQR), 10^9^/L	8.56 (6.39–11.46)	8.10 (6.80–10.22)		0.50
Neutrophile granulocyte count, median (IQR), 10^9^/L	6.36 (4.09–9.55)	5.82 (3.99–7.51)		0.12
Lymphocyte count, median (IQR), 10^9^/L	1.42 (0.95–1.96)	1.60 (1.10–2.26)		0.048
Neutrophil to lymphocyte ratio, median (IQR)	4.32 (2.44–8.46)	3.29 (1.72–5.59)		0.02
BNP, median (IQR), pg/ml	55.60 (32.63–105.15)	66.30 (31.35–110.38)		0.78

APT, antiplatelet therapy; APTT, activated partial thromboplastin time; BNP, B-type natriuretic peptide; CRP, C-reactive protein; GCS, Glasgow coma scale; HE, hematoma expansion; ICH, intracerebral hemorrhage; IQR, interquartile range; IVH, intraventricular hemorrhage; MLS, midline shift; NCCT, noncontrast computed tomography; PT-INR, prothrombin time/international normalized ratio

**Table 3:** Univariate analysis of clinical baseline characteristics in the two lobar ICH cohorts

Variable	Non-HE (*n* = 93)	HE (*n* = 27)	*P* value
Male, *n* (%)	58 (62.4)	19 (70.4)	0.45
Age, median (IQR), y	70.00 (63.00–81.00)	73.00 (68.00–85.00)	0.10
Time from onset to NCCT, median (IQR), h	4.28 (2.16–8.93)	4.50 (1.90–9.08)	0.94
Admission GCS score, median (IQR)	14.00 (11.00–15.00)	13.00 (10.00–15.00)	0.65
van Swieten scale, median (IQR)	1.00 (0.00–3.00)	1.00 (0.00–2.00)	0.95
van Swieten scale > 1, *n* (%)	38 (40.9)	12 (44.4)	0.74
Admission systolic pressure, mean ± SD, mmhg	169.24 ± 24.85	172.44 ± 23.07	0.55
Admission diastolic pressure, median (IQR), mmhg	102.00 (83.50–111.50)	90.00 (80.00–110.00)	0.18
Smoking, *n* (%)	25 (26.9)	4 (14.8)	0.20
Drinking, *n* (%)	15 (16.1)	4 (14.8)	0.87
Diabetes, *n* (%)	16 (17.2)	9 (33.3)	0.07
Coronary sclerosis, *n* (%)	63 (67.7)	19 (70.4)	0.80
Atrial fibrillation, *n* (%)	9 (9.7)	1 (3.7)	0.55
Hypertension, *n* (%)	69 (74.2)	20 (74.1)	0.99
Stroke, *n* (%)	21 (22.6)	5 (18.5)	0.65
Anticoagulant therapy, *n* (%)	18 (19.4)	5 (18.5)	0.92
Hemostatic therapy, *n* (%)	67 (72.0)	23 (85.2)	0.17
APT, *n* (%)	20 (21.5)	3 (11.1)	0.23
Subarachnoid hemorrhage, *n* (%)	35 (37.6)	16 (59.3)	0.045
Fluid level, *n* (%)	17 (18.3)	6 (22.2)	0.65
Irregular shape, *n* (%)	60 (64.5)	24 (88.9)	0.02
Hypodensities, *n* (%)	35 (37.6)	13 (48.1)	0.33
Blend sign, *n* (%)	37 (39.8)	17 (63.0)	0.03
Island sign, *n* (%)	25 (26.9)	9 (33.3)	0.51
Black hole sign, *n* (%)	17 (18.3)	4 (14.8)	0.90
MLS, *n* (%)	17 (18.3)	6 (22.2)	0.65
IVH, *n* (%)	13 (14.0)	8 (29.6)	0.11
Volume, median (IQR), ml	19.13 (7.22–34.72)	30.12 (12.70–45.05)	0.03
Density, mean ± SD, hu	58.66 ± 5.63	58.61 ± 4.91	0.96
Rate of bleeding, median (IQR), ml/h	3.69 (1.28–9.90)	7.92 (2.36–11.65)	0.06
Creatine kinase, median (IQR), U/L	90.50 (54.25–137.25)	81.00 (56.00–118.00)	0.70
Blood glucose, median (IQR), mmol/L	7.23 (6.17–10.05)	6.74 (6.18–8.44)	0.60
Fibrinogen, median (IQR), g/L	2.84 (2.37–3.36)	2.38 (2.13–2.85)	0.01
D-dimer, median (IQR), mg/L	0.55 (0.32–1.10)	0.55 (0.41–1.26)	0.50
APTT, mean ± SD, s	27.52 ± 2.80	27.15 ± 1.95	0.52
PT-INR, median (IQR)	0.95 (0.92–1.03)	0.95 (0.91–0.98)	0.48
CRP, median (IQR), mg/L	4.00 (1.16–9.80)	2.10 (0.69–5.10)	0.15
Leukocyte count, median (IQR), 10^9^/L	9.08 (7.07–10.88)	7.65 (6.83–9.36)	0.06
Neutrophile granulocyte count, median (IQR), 10^9^/L	6.71 (5.10–9.37)	5.40 (4.36–6.93)	0.01
Lymphocyte count, median (IQR), 10^9^/L	1.21 (0.87–1.74)	1.46 (0.93–2.34)	0.11
NLR, median (IQR)	5.63 (3.27–9.62)	3.19 (2.30–6.31)	0.01
BNP, median (IQR), pg/ml	68.90 (44.15–150.70)	66.30 (43.70–166.20)	0.89

APT, antiplatelet therapy; APTT, activated partial thromboplastin time; BNP, B-type natriuretic peptide; CRP, C-reactive protein; GCS, Glasgow coma scale; HE, hematoma expansion; ICH, intracerebral hemorrhage; IQR, interquartile range; IVH, intraventricular hemorrhage; MLS, midline shift; NCCT, noncontrast computed tomography; NLR, neutrophil-to-lymphocyte ratio; PT-INR, prothrombin time/international normalized ratio

In this study, the neutrophil count was significantly higher in the non-HE group compared to the HE group, and the lymphocyte count was significantly lower, leading to a higher NLR in the non-HE group. We noted that in previous studies, higher neutrophil counts, lower lymphocyte counts, and a higher NLR were associated with poorer 3-month outcomes [14]. We conducted a supplementary analysis to confirm this, and the results were consistent with previous studies, although the differences were not significant, as shown in Supplementary Table 5.

### Predictors of HE

**Fig. 2:**
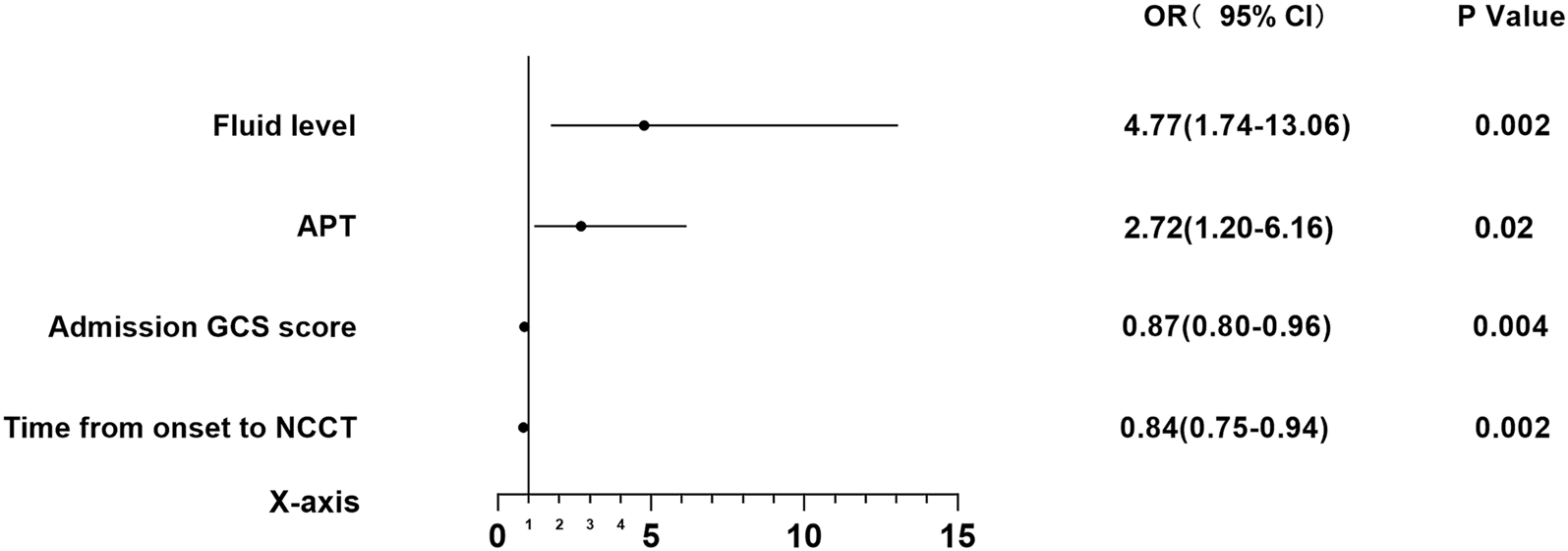
Forest plot presenting the OR of hematoma expansion among patients with deep intracerebral hemorrhage. APT, antiplatelet therapy; CI, confidence interval; GCS glasgow coma scale; NCCT, noncontrast computed tomography; OR odds ratio

**Fig. 3:**
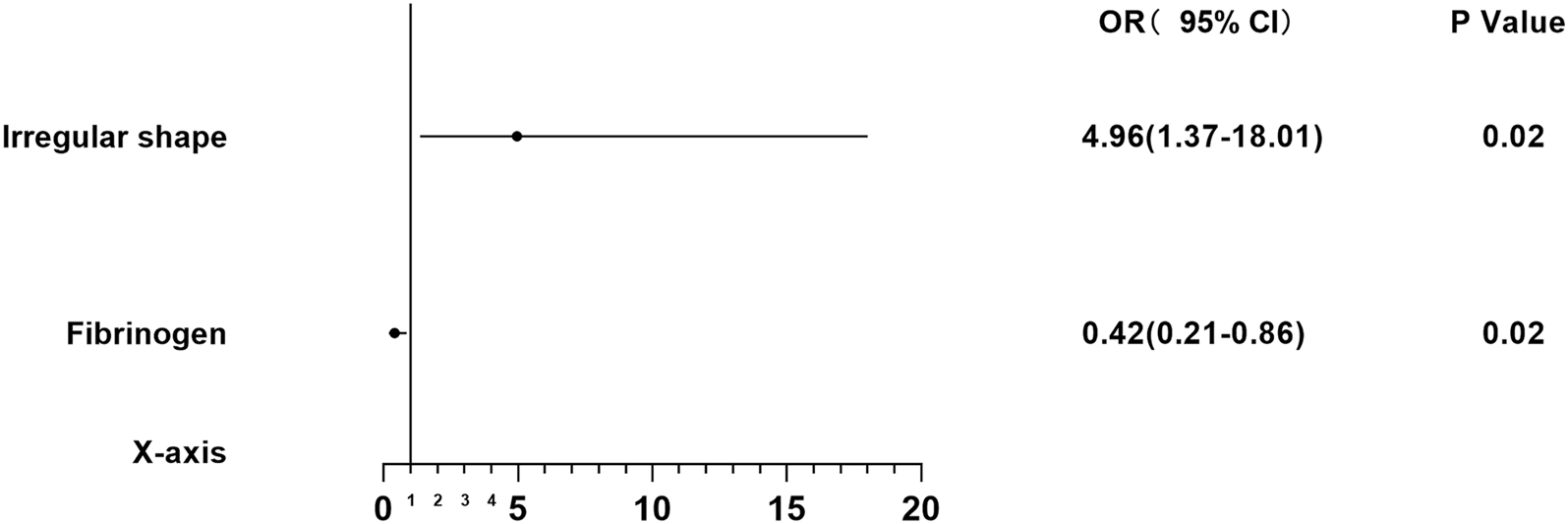
Forest plot presenting the OR of hematoma expansion among patients with lobar intracerebral hemorrhage. CI confidence interval; OR odds ratio

Variables with a *P* value < 0.05 in the difference analysis were included in the binary logistic regression analysis for HE. Before constructing the logistic regression model, the selected variables underwent multicollinearity diagnostics, and all had variance inflation factor values less than 10, indicating no multicollinearity. We adjusted for the rate of bleeding in the deep ICH group and the time from onset to NCCT in the lobar ICH group. After Bonferroni correction, the statistical significance threshold was set at *P* = 0.01 for the deep ICH group and *P* = 0.03 for the lobar ICH group. Table 4 lists all the variables included in the final logistic regression model of ICH cohorts. In the deep ICH cohort, the time from symptom onset to NCCT examination (odds ratio [OR] 0.84; 95% confidence interval [CI] 0.75–0.94; *P* = 0.002), admission GCS score (OR 0.87; 95% CI 0.80–0.96; *P* = 0.004), and fluid level (OR 4.77; 95% CI 1.74–13.06; *P* = 0.002) were independent risk factors for HE. Adjusting for the rate of bleeding had no impact on the results. In the lobar ICH cohort, fibrinogen (OR 0.42; 95% CI 0.21–0.86; *P* = 0.02) and irregular shape (OR 4.96; 95% CI 1.37–18.01; *P* = 0.02) were independent risk factors for HE. Adjusting for the time from onset to NCCT had minimal impact on the results. The Hosmer–Lemeshow goodness-of-fit test showed no statistically significant difference between predicted and observed values, indicating that the model fit well in the deep ICH group (*P* = 0.67) and the lobar ICH group (*P* = 0.98). Figures 2 and 3 illustrate the relationships between binary logistic regression variables and HE in the deep ICH cohort and the lobar ICH cohort.

**Table 4:** Multivariable-adjusted regression analysis results

Variable	Unadjusted OR (95% CI)	*P* value	Adjusted OR (95% CI)	*P* value
Deep ICH^a^				
Time from onset to NCCT	0.84 (0.75–0.94)	0.002	0.84 (0.75–0.94)	0.002
Admission GCS score	0.87 (0.80–0.96)	0.004	0.87 (0.80–0.96)	0.004
APT	2.72 (1.20–6.16)	0.02	2.72 (1.20–6.16)	0.02
Fluid level	4.77 (1.74–13.06)	0.002	4.77 (1.74–13.06)	0.002
Lobe ICH^b^				
Fibrinogen	0.42 (0.21–0.86)	0.02	0.42 (0.21–0.86)	0.02
Irregular shape	4.83 (1.33–17.54)	0.02	4.96 (1.37–18.01)	0.02

APT, antiplatelet therapy; CI, confidence interval; GCS, Glasgow coma scale; ICH, intracerebral hemorrhage; NCCT, noncontrast computed tomography; OR, odds ratio

^a^Adjusted for rate of bleeding

^b^Adjusted for time from onset to NCCT

## Discussion

Previous studies have indicated that deep ICH and lobar ICH differ in terms of clinical and radiological characteristics, incidence of HE, and prognosis [11, 12]. We further stratified deep ICH and lobar ICH into two subgroups, effectively controlling for the confounding effect of location, and identified distinct risk factors for HE in each group. When constructing predictive tools for HE using these risk factors, differentiation based on the site of hemorrhage should be considered.

This study defined HE as “an absolute increase in hematoma volume > 6 mL or a relative increase in volume from baseline to follow-up NCCT > 33%” and found no significant difference in the incidence of HE between deep and lobar ICH, consistent with previous research conclusions [10, 11]. However, our findings contradict those of Kuohn et al. [12], which may be attributed to differences in the standardization of the time from onset to NCCT and the definition of HE. Our study aligns with the research conducted by Roh et al. [10, 11]. In the differential analysis of clinical and radiological characteristics between deep ICH and lobar ICH, we not only identified clinical feature discrepancies consistent with previous studies, such as age, blood pressure, history of coronary heart disease, baseline hematoma volume, midline shift, and intraventricular hemorrhage [10–12], but also found that patients with lobar ICH had higher van Swieten scale scores compared to those with deep ICH, indicating more severe leukoaraiosis in lobar ICH. However, in subsequent subgroup multivariate analyses, we did not find a significant association with HE, which is in agreement with previous findings [15]. This suggests that the van Swieten scale is not effective for predicting HE. Our research also revealed that subarachnoid hemorrhage, fluid-level signs, irregular signs, blend signs, and island signs were more common in lobar ICH. Because of the superficial location of lobar ICH, it is more likely to be accompanied by subarachnoid hemorrhage. Given the differences in etiology, brain tissue and nuclear structure, and age at onset between patients with lobar and those with deep ICH, the reasons for the disparities in imaging markers such as fluid-level signs, irregular signs, mixed signs, and island signs may be multifactorial and require further investigation to elucidate the underlying mechanisms.

In this study, after Bonferroni correction, fluid level, admission GCS score, and time from onset to NCCT were independently predictive of HE in patients with deep ICH. Previous research has found that fluid level can be used to predict HE, and a interrater agreement analysis for fluid level achieved a consistency coefficient of 0.89, indicating its stability for HE prediction [16, 17]. Research has suggested that fluid level reflects abnormal coagulation within the hemorrhage, leading to early precipitation of higher density proteins [18]. In our study, fluid levels were significantly more prevalent in patients with lobar ICH than in patients with deep ICH, yet the correlation between fluid levels and HE was stronger in patients with deep ICH. Whether this is related to functional coagulation differences in different brain regions remains to be further investigated. Additionally, it is worth noting that this study included patients with ICH who had received anticoagulant therapy, and all patients were treated according to the anticoagulant reversal protocols recommended by the Chinese Cerebrovascular Disease Clinical Management Guidelines, based on the type of oral anticoagulant and INR values. As shown in Supplementary Table 6–8, we explored the relationship between anticoagulant therapy and fluid levels in the overall ICH, deep ICH, and lobar ICH cohorts and found that patients with ICH who had received anticoagulant therapy were more likely to have fluid levels, although the results were not statistically significant. In this study, the OR for the presence of fluid levels was 4.77, indicating that deep ICH with fluid levels was associated with a 4.77-fold increased risk of HE compared to deep ICH without fluid levels.

In this study, we defined the time from onset to NCCT as within 24 h; however, the majority of patients (74%) had a time from onset to NCCT of less than 6 h. As depicted in Supplementary Table 9, to investigate the impact of time from onset to NCCT on the incidence of HE, we analyzed the incidence of HE in each cohort when time from onset to NCCT was less than 6 h and less than 24 h. We found that when time from onset to NCCT was less than 6 h, there was a slight increase in the incidence of HE, with no significant difference observed between the deep ICH and lobar ICH groups. Because HE predominantly occurs within the first 3–6 h after onset [2], extending the time window beyond 6 h makes it difficult to capture HE occurrences. Adjusting for the rate of bleeding had no effect on the regression results, suggesting that the rate of bleeding may simply be an associated phenomenon of a shorter time from onset to NCCT.

The admission GCS score reflects the level of consciousness in patients, with lower scores indicating more severe coma. As seen in Table 1 comparing baseline characteristics of ICH at different locations, the admission GCS scores were significantly lower in the deep ICH cohort (median 12.00; IQR 9.00–13.00) compared to the lobar ICH cohort (median 14.00; IQR 11.00–15.00). Deep ICH typically involves neuro-nuclei, with worse consciousness levels indicating more severe bleeding and localized brain injury, thus increasing the likelihood of HE. In the lobar ICH cohort, there were no differences in GCS scores between groups, suggesting that GCS score may be a specific predictor of HE for deep ICH. Research by Morotti et al. has shown that admission GCS score and time from onset to NCCT are independent predictors of severe HE [8].

In the lobar ICH cohort, after the Bonferroni correction, irregular shape of the hematoma and fibrinogen level independently predicted the occurrence of HE. Previous studies have suggested that an irregular hematoma shape may represent secondary HE, with continuous bleeding and increased hemorrhagic pressure forcing the hematoma to expand into surrounding brain tissue. Vascular rupture under pressure shear leads to hematoma spread, losing the regular elliptical structure [18]. Low fibrinogen levels in the blood hinder primary and secondary coagulation processes, increasing the risk of HE [19]. Normally, fibrinogen levels increase with age [20]. In this study, the lobar ICH group (median 71.00 years; IQR 64.00–84.00 years) was significantly older than the deep ICH group (median 64.00 years; IQR 53.00–71.00 years), but the fibrinogen (g/L) level in the lobar ICH group (median 2.62; IQR 2.29–3.31) was lower than that in the deep ICH group (median 2.65; IQR 2.27–3.17), which may be related to hemostatic treatment and the use of antiplatelet medications such as aspirin and clopidogrel.

In the study by Lattanzi et al., higher neutrophil counts, lower lymphocyte counts, and a higher NLR were associated with poorer 3-month outcomes [14]. Supplementary Table 5 demonstrates a differential analysis of neutrophil, lymphocyte, and NLR levels between patients with good and poor prognosis, which is consistent with previous research. However, because of inconsistencies in previous studies, the relationship between NLR and HE remains controversial. In our analysis of HE, compared to the HE group, the non-HE group exhibited higher neutrophil counts, lower lymphocyte counts, and a higher NLR. In contrast, studies by Alimohammadi et al. and Kim et al. found that the HE group had higher neutrophil counts, lower lymphocyte counts, and a higher NLR, with the NLR being an independent predictor of HE [6, 21]. Conversely, Fonseca et al. reported that the HE group had lower neutrophil counts, lower lymphocyte counts, and a lower NLR, which aligns with our findings [22]. A meta-analysis by Shi et al. suggested no significant association between the NLR and HE [23]. To clarify the reasons for the discrepancies in these studies, it is necessary to analyze neutrophils and lymphocytes separately. Although there are conflicting results in the studies examining the NLR and HE, it is consistent across various studies that both the HE group and the group with poor prognosis have lower lymphocyte levels. A study by Mao et al. found that adoptive transfer of regulatory T cells in a model of autologous ICH can mitigate inflammatory responses, improve brain barrier integrity, reduce cerebral edema, and decrease neuronal death [24]. This could explain why reduced lymphocyte counts are significantly associated with poor prognosis and HE. The main contradiction lies in the inconsistent relationships between increased neutrophil counts and HE across different studies. Most research focuses on the inflammatory response’s effects on the blood–brain barrier, vascular permeability, and neuronal cells. However, inflammation also has a more direct effect on promoting coagulation and thrombosis. A study by Steppich et al. showed that neutrophil-activated proteases mediate endothelial cell damage and procoagulant responses [25]. There is a complex interplay between inflammation and hemostasis, involving proinflammatory cytokines, chemokines, adhesion molecules, tissue factor expression, platelet and endothelial activation, and microparticles. Inflammation increases procoagulant factors and also suppresses natural anticoagulant pathways and fibrinolytic activity, leading to a thrombotic tendency [26]. On the other hand, thrombin-induced secretion of proinflammatory cytokines and growth factors can exacerbate the inflammatory response, creating a vicious cycle. Further research is needed to elucidate the mechanisms by which neutrophil-mediated inflammatory injury and procoagulant effects influence functional outcomes and HE in ICH.

This study also has some limitations. Firstly, it is a retrospective study, which inherently involves some selection bias that is difficult to avoid. Secondly, in this study, the window for the first CT scan was defined as within 24 h, whereas HE predominantly occurs in the early stages of cerebral hemorrhage (3–6 h). This may result in some cases (26%) not capturing the progression of HE because of delayed CT acquisition. However, in our supplementary analyses, the incidence of HE did not vary significantly. Additionally, the follow-up CT scan was set to a 72-h window. As depicted in Supplementary Table 10, we also conducted a supplementary analysis, which showed an increased incidence of HE when the window was reduced to 48 h. It is important to note that HE primarily occurs in the early stages, and controlling the window for the first CT scan ensures capturing the pre-progression state of the hematoma, which directly affects the observation of HE on subsequent follow-up CT scans. However, extending the follow-up CT window does not alter the established fact of HE. The increased incidence of HE observed with a shortened follow-up window may be attributed to two factors: first, the inclusion of patients with a lower probability of HE in the extended time window, which reduces the overall incidence of HE, and second, in clinical practice, if patients do not show progression or sudden symptoms after admission, the follow-up CT scan is typically performed between 48 and 72 h post admission, indicating that these patients are mostly in an improving condition without HE. In research, the exclusion of these patients may not reflect the true incidence of HE, and careful consideration is required to decide whether they should be included in the study population. Lastly, the analysis is based on data from a single medical center, and the results need to be further validated.

## Conclusions

Fluid level, admission GCS score, and shorter time from onset to NCCT are independent predictors of HE in deep ICH, whereas irregular shape and low fibrinogen levels are independent predictors of HE in lobar ICH. This study demonstrates that the risk factors for HE differed between deep and lobar ICH, which is of great significance for exploring the mechanisms of HE in different ICH locations and constructing precise HE prediction models.

## Supplementary Information

Below is the link to the electronic supplementary material.

Supplementary file1 (DOCX 17 KB)

## Data Availability

Anonymized data can be made available by request from any qualified investigator.

## References

[1] Wang W, Jiang B, Sun H, et al. Prevalence, incidence, and mortality of stroke in China: results from a nationwide population-based survey of 480 687 adults. Circulation. 2017;135(8):759–71. 10.1161/circulationaha.116.025250. (**In eng**). 28052979 10.1161/CIRCULATIONAHA.116.025250

[2] Morotti A, Boulouis G, Dowlatshahi D, et al. Intracerebral haemorrhage expansion: definitions, predictors, and prevention. Lancet Neurol. 2023;22(2):159–71. 10.1016/s1474-4422(22)00338-6. (**In eng**). 36309041 10.1016/S1474-4422(22)00338-6

[3] Seiffge DJ, Polymeris AA, Law ZK, et al. Cerebral amyloid angiopathy and the risk of hematoma expansion. Ann Neurol. 2022;92(6):921–30. 10.1002/ana.26481. (**In eng**). 36054211 10.1002/ana.26481 PMC9804369

[4] Morotti A, Boulouis G, Charidimou A, et al. Imaging markers of intracerebral hemorrhage expansion in patients with unclear symptom onset. Int J Stroke Off J Int Stroke Soc. 2022;17(9):1013–20. 10.1177/17474930211068662. (**In eng**). 10.1177/17474930211068662 35318878

[5] Morotti A, Arba F, Boulouis G, Charidimou A. Noncontrast CT markers of intracerebral hemorrhage expansion and poor outcome: a meta-analysis. Neurology. 2020;95(14):632–43. 10.1212/wnl.0000000000010660. (**In eng**). 32847959 10.1212/WNL.0000000000010660

[6] Kim Y, Sohn JH, Kim C, Park SY, Lee SH. The clinical value of neutrophil-to-lymphocyte ratio and platelet-to-lymphocyte ratio for predicting hematoma expansion and poor outcomes in patients with acute intracerebral hemorrhage. J Clin Med. 2023;12(8):3004. 10.3390/jcm12083004. (**In eng**). 37109337 10.3390/jcm12083004 PMC10145379

[7] Cappellari M, Zivelonghi C, Moretto G, et al. The etiologic subtype of intracerebral hemorrhage may influence the risk of significant hematoma expansion. J Neurol Sci. 2015;359(1–2):293–7. 10.1016/j.jns.2015.11.024. (**In eng**). 26671130 10.1016/j.jns.2015.11.024

[8] Morotti A, Li Q, Nawabi J, et al. Predictors of severe intracerebral hemorrhage expansion. Eur Stroke J. 2024;9(3):623–9. 10.1177/23969873241247436. (**In eng**). 38627953 10.1177/23969873241247436 PMC11418511

[9] Morotti A, Poli L, Leuci E, et al. Subarachnoid extension predicts lobar intracerebral hemorrhage expansion. Stroke. 2020;51(5):1470–6. 10.1161/strokeaha.119.028338. (**In eng**). 32200757 10.1161/STROKEAHA.119.028338

[10] Roh D, Boehme A, Young C, et al. Hematoma expansion is more frequent in deep than lobar intracerebral hemorrhage. Neurology. 2020;95(24):e3386–93. 10.1212/wnl.0000000000010990. (**In eng**). 33219144 10.1212/WNL.0000000000010990 PMC7836660

[11] Roh D, Sun CH, Murthy S, et al. Hematoma expansion differences in lobar and deep primary intracerebral hemorrhage. Neurocrit Care. 2019;31(1):40–5. 10.1007/s12028-018-00668-2. (**In eng**). 30756318 10.1007/s12028-018-00668-2 PMC6609462

[12] Kuohn LR, Witsch J, Steiner T, et al. Early deterioration, hematoma expansion, and outcomes in deep versus lobar intracerebral hemorrhage: the FAST trial. Stroke. 2022;53(8):2441–8. 10.1161/strokeaha.121.037974. (**In eng**). 35360929 10.1161/STROKEAHA.121.037974

[13] Fedorov A, Beichel R, Kalpathy-Cramer J, et al. 3D slicer as an image computing platform for the quantitative imaging network. Magn Resonance Imaging. 2012;30(9):1323–41. 10.1016/j.mri.2012.05.001. (**In eng**). 10.1016/j.mri.2012.05.001 PMC3466397 22770690

[14] Lattanzi S, Cagnetti C, Provinciali L, Silvestrini M. Neutrophil-to-lymphocyte ratio predicts the outcome of acute intracerebral hemorrhage. Stroke. 2016;47(6):1654–7. 10.1161/strokeaha.116.013627. (**In eng**). 27165957 10.1161/STROKEAHA.116.013627

[15] Hansen BM, Ullman N, Muschelli J, et al. Relationship of white matter lesions with intracerebral hemorrhage expansion and functional outcome: MISTIE II and CLEAR III. Neurocrit Care. 2020;33(2):516–24. 10.1007/s12028-020-00916-4. (**In eng**). 32026447 10.1007/s12028-020-00916-4 PMC7416541

[16] Nehme A, Ducroux C, Panzini MA, et al. Non-contrast CT markers of intracerebral hematoma expansion: a reliability study. Eur Radiol. 2022;32(9):6126–35. 10.1007/s00330-022-08710-w. (**In eng**). 35348859 10.1007/s00330-022-08710-w

[17] Blacquiere D, Demchuk AM, Al-Hazzaa M, et al. Intracerebral hematoma morphologic appearance on noncontrast computed tomography predicts significant hematoma expansion. Stroke. 2015;46(11):3111–6. 10.1161/strokeaha.115.010566. (**In eng**). 26451019 10.1161/STROKEAHA.115.010566

[18] Boulouis G, Morotti A, Charidimou A, Dowlatshahi D, Goldstein JN. noncontrast computed tomography markers of intracerebral hemorrhage expansion. Stroke. 2017;48(4):1120–5. 10.1161/strokeaha.116.015062. (**In eng**). 28289239 10.1161/STROKEAHA.116.015062 PMC5378158

[19] Wang G, Zhang J. Hematoma expansion: clinical and molecular predictors and corresponding pharmacological treatment. Curr Drug Targets. 2017;18(12):1367–76. 10.2174/1389450117666160712092224. (**In eng**). 27411712 10.2174/1389450117666160712092224

[20] Folsom AR. Epidemiology of fibrinogen. Eur Heart J. 1995;16(suppl A):21–4. 10.1093/eurheartj/16.suppl_A.21. (**In eng**). 10.1093/eurheartj/16.suppl_a.21 7796826

[21] Alimohammadi E, Bagheri SR, Mardanpour P, Moradi F, Arjmandnia F, Esmaeili N. Baseline neutrophil-lymphocyte ratio can be associated with hematoma expansion in patients with intracerebral hemorrhage: a retrospective observational study. BMC Neurosci. 2022;23(1):18. 10.1186/s12868-022-00705-z. (**In eng**). 35337267 10.1186/s12868-022-00705-z PMC8957183

[22] Fonseca S, Costa F, Seabra M, et al. Systemic inflammation status at admission affects the outcome of intracerebral hemorrhage by increasing perihematomal edema but not the hematoma growth. Acta Neurol Belg. 2021;121(3):649–59. 10.1007/s13760-019-01269-2. (**In eng**). 31912444 10.1007/s13760-019-01269-2

[23] Shi M, Li XF, Zhang TB, Tang QW, Peng M, Zhao WY. Prognostic role of the neutrophil-to-lymphocyte ratio in intracerebral hemorrhage: a systematic review and meta-analysis. Front Neurosci. 2022;16:825859. 10.3389/fnins.2022.825859. (**In eng**). 35360156 10.3389/fnins.2022.825859 PMC8960242

[24] Mao LL, Yuan H, Wang WW, et al. Adoptive regulatory T-cell therapy attenuates perihematomal inflammation in a mouse model of experimental intracerebral hemorrhage. Cell Mol Neurobiol. 2017;37(5):919–29. 10.1007/s10571-016-0429-1. (**In eng**). 27678140 10.1007/s10571-016-0429-1 PMC11482213

[25] Steppich BA, Seitz I, Busch G, Stein A, Ott I. Modulation of tissue factor and tissue factor pathway inhibitor-1 by neutrophil proteases. Thromb Haemost. 2008;100(6):1068–75 (**In eng**). 19132232

[26] Aksu K, Donmez A, Keser G. Inflammation-induced thrombosis: mechanisms, disease associations and management. Curr Pharma Des. 2012;18(11):1478–93. 10.2174/138161212799504731. (**In eng**). 10.2174/138161212799504731 22364132

