# Supplementary material for: Differential Risk Factors for Hematoma Expansion in Deep and Lobar Intracerebral Hemorrhage
Source: Neurocrit Care. 2025 Feb 4;43(1):80–90. doi: 10.1007/s12028-025-02218-z (PMC12321654; doi:10.1007/s12028-025-02218-z)
Supplement: Supplementary file 1 — Supplementary file1 (DOCX 17 KB) [file 12028_2025_2218_MOESM1_ESM.docx]

| Table 5 Results of differential analysis of neutrophils and lymphocytes in prognostic subgroups | | | | |
| --- | --- | --- | --- | --- |
| Variable | Good outcome (n=240) | | Poor outcome (n=164) | *P* Value |
| Neutrophile granulocyte | 5.99(4.35-8.30) | 6.66(4.51-9.53) | | 0.07 |
| Lymphocyte | 1.45(1.02-2.00) | 1.39(0.83-1.90) | | 0.17 |
| NLR | 3.82(2.41-7.52) | 4.64(2.44-9.97) | | 0.07 |

Abbreviations: NLR: neutrophil-to-lymphocyte ratio

| Table 6 Correlation analysis between anticoagulant therapy and fluid level of overall ICH cohort | | | |
| --- | --- | --- | --- |
| Variable | Non-anticoagulant therapy (n=319) | Anticoagulant therapy (n=85) | *P* Value |
| Fluid level | 33(10.3) | 12(14.1) | 0.33 |

Abbreviations: ICH: intracerebral hemorrhage

| Table 7 Correlation analysis between anticoagulant therapy and fluid level of deep ICH cohort | | | |
| --- | --- | --- | --- |
| Variable | Non-anticoagulant therapy (n=221) | Anticoagulant therapy (n=63) | *P* Value |
| Fluid level | 17(7.7) | 5(7.9) | 0.95 |

Abbreviations: ICH: intracerebral hemorrhage

| Table 8 Correlation analysis between anticoagulant therapy and fluid level of lobar ICH cohort | | | |
| --- | --- | --- | --- |
| Variable | Non-anticoagulant therapy (n=98) | Anticoagulant therapy (n=22) | *P* Value |
| Fluid level | 16(16.3) | 7(31.8) | 0.17 |

Abbreviations: ICH: intracerebral hemorrhage

| Table 9 Sensitivity analysis of "Time from onset to NCCT" | | | |  |
| --- | --- | --- | --- | --- |
| Variable | All ICH (n=300) | Lobe ICH (n=76) | Deep ICH (n=224) | *P* Value |
| Time from onset to NCCT﹤6h | | | | |
| HE(﹥6ml or 33%) | 78(26.0) | 18(23.7) | 60(26.8) | 0.59 |
| Variable | All ICH (n=404) | Lobe ICH (n=120) | Deep ICH (n=284) | *P* Value |
| Time from onset to NCCT﹤24h | | | | |
| HE(﹥6ml or 33%) | 93(23.0) | 27(22.5) | 66(23.2) | 0.87 |

Abbreviations: NCCT: non-contrast CT; ICH: intracerebral hemorrhage; HE: hematoma expansion

| Table 10 Sensitivity analysis of "Time from onset to follow up NCCT" | | | |  |
| --- | --- | --- | --- | --- |
| Variable | All ICH (n=247) | Lobe ICH (n=66) | Deep ICH (n=181) | *P* Value |
| Time from onset to follow up NCCT﹤48h | | | | |
| HE(﹥6ml or 33%) | 66(26.7) | 20(30.3) | 46(25.4) | 0.44 |
| Variable | All ICH (n=404) | Lobe ICH (n=120) | Deep ICH (n=284) | *P* Value |
| Time from onset to follow up NCCT﹤72h | | | | |
| HE(﹥6ml or 33%) | 93(23.0) | 27(22.5) | 66(23.2) | 0.87 |

Abbreviations: NCCT: non-contrast CT; ICH: intracerebral hemorrhage; HE: hematoma expansion
